# Ionic Extracts of Magnesium Powders Promote In Vitro Lymphangiogenesis

**DOI:** 10.3390/biomedicines14040913

**Published:** 2026-04-16

**Authors:** Yan Wang, Xiran Liu, Zerui Shan, Yu Xia, Yiya Weng, Magdalena M. Stevanović, Nenad Filipović, Kai Zheng, Junqing Ma

**Affiliations:** 1 State Key Laboratory Cultivation Base of Research, Prevention and Treatment for Oral Diseases, Nanjing Medical University, Nanjing 210029, China; dent_wy@163.com (Y.W.); shanzerui_njmu@126.com (Z.S.); yuukaxia_njmu@163.com (Y.X.); wengyiya@163.com (Y.W.); 2 Stomatological College, Nanjing Medical University, Nanjing 210029, China; xiranliu@stu.njmu.edu.cn; 3 Jiangsu Province Engineering Research Center of Stomatological Translational Medicine, Nanjing Medical University, Nanjing 210029, China; 4 Group for Biomedical Engineering and Nanobiotechnology, Institute of Technical Sciences of the Serbian Academy of Sciences and Arts, 11000 Belgrade, Serbia; magdalena.stevanovic@itn.sanu.ac.rs (M.M.S.); nenad.filipovic@itn.sanu.ac.rs (N.F.); 5 Savaid Stomatology School, Hangzhou Medical College, Hangzhou 311399, China

**Keywords:** magnesium, ion release, lymphangiogenesis, migration, tube formation

## Abstract

**Background/Objectives:** Biodegradable magnesium (Mg)-based biomaterials release Mg^2+^ ions during degradation and may promote vascular-related regeneration. However, their effects on lymphatic endothelial cells (LECs) and lymphangiogenesis remain unclear. This study investigated whether magnesium powder-derived ionic extracts could enhance lymphangiogenesis-related behaviors of LECs in vitro. **Methods:** Mg powder extracts were prepared and diluted for in vitro treatment. After viability screening, Mg (1:10), Mg (1:100), and Mg (1:1000) were selected for further analysis. LEC proliferation, migration, and tube formation were assessed, together with intracellular reactive oxygen species (ROS) levels and the expression of VEGFA, VEGFC, and VEGFR3. **Results:** Mg (1:10) and Mg (1:100) showed good cytocompatibility and significantly promoted LEC proliferation, migration, and tube formation compared with the control and Mg (1:1000) groups. These effects were accompanied by reduced intracellular ROS levels and increased expression of VEGFA, VEGFC, and VEGFR3. **Conclusions:** Magnesium powder-derived ionic extracts enhance lymphangiogenesis-related responses of LECs in vitro, particularly at the 1:10 and 1:100 dilutions. These findings support the potential of Mg-based biodegradable biomaterials for lymphatic tissue regeneration.

## 1. Introduction

Severe tissue injuries, including extensive burns, chronic wounds, and especially oncologic resection leading to secondary lymphedema, remain major unsolved challenges in regenerative medicine [1,2,3], severely compromising patients’ quality of life and imposing substantial global healthcare burdens [4]. The lymphatic system plays an indispensable role in tissue repair by regulating interstitial fluid balance, facilitating immune surveillance, transporting lipids, and resolving inflammation [5]. Lymphangiogenesis, the sprouting and remodeling of new lymphatic vessels, is therefore critical for restoring tissue homeostasis following injury or disease [6]. However, the endogenous regenerative capacity of lymphatic vessels is typically limited, particularly in severe pathological contexts, resulting in persistent dysfunction and impaired healing.

Lymphatic endothelial cells (LECs) are structurally and functionally distinct from blood endothelial cells (BECs). LECs are characterized by the expression of specific molecular markers such as PROX1 and VEGFR3, which play critical roles in lymphatic development and maintenance. In contrast to angiogenesis, lymphangiogenesis is regulated by distinct signaling pathways and serves specialized functions in tissue fluid homeostasis, immune cell trafficking, and lipid absorption [7,8]. Current therapeutic strategies to stimulate lymphangiogenesis primarily involve the administration of recombinant lymphangiogenic growth factors, notably vascular endothelial growth factor-C (VEGF-C) and VEGF-D [9,10,11], or cell-based approaches such as endothelial progenitor cell transplantation [12,13]. While these methods have shown promise, they are constrained by limitations including poor protein stability, high production costs, potential immunogenicity, and ethical/regulatory hurdles associated with cell therapies. Consequently, there is growing interest in bioactive, biodegradable biomaterials that can intrinsically promote lymphatic regeneration, offering a potentially safer, cost-effective, and more translatable alternative [14,15,16].

Among biodegradable materials, magnesium (Mg) and its alloys stand out due to their favorable biocompatibility, appropriate mechanical strength, and controlled degradation profile in physiological environments [17,18,19,20,21]. Released Mg^2+^ ions serve as essential cofactors for numerous enzymes (e.g., ATPases) and participate in critical cellular processes, including energy metabolism, nucleic acid synthesis, and protein production [22,23]. Substantial evidence demonstrates that Mg^2+^ enhances angiogenesis by modulating vascular endothelial cell functions, such as proliferation, migration, and tube formation [24,25,26]. Given the shared developmental origins, structural analogies, and overlapping signaling pathways between blood vascular and lymphatic endothelial cells, it is plausible that Mg^2+^ may similarly benefit lymphatic endothelial cell (LEC) behavior and promote lymphangiogenesis. Mechanistically, Mg^2+^ regulates intracellular Ca^2+^ homeostasis and influences key pro-regenerative pathways, including PI3K/Akt and MAPK/ERK signaling, which govern cell survival, proliferation, migration, and differentiation [24,27,28,29]. Despite these parallels, the specific effects of Mg^2+^ on LEC and its capacity to drive lymphatic vessel regeneration remain largely uninvestigated, constituting a significant knowledge gap in the field.

**Scheme 1:**
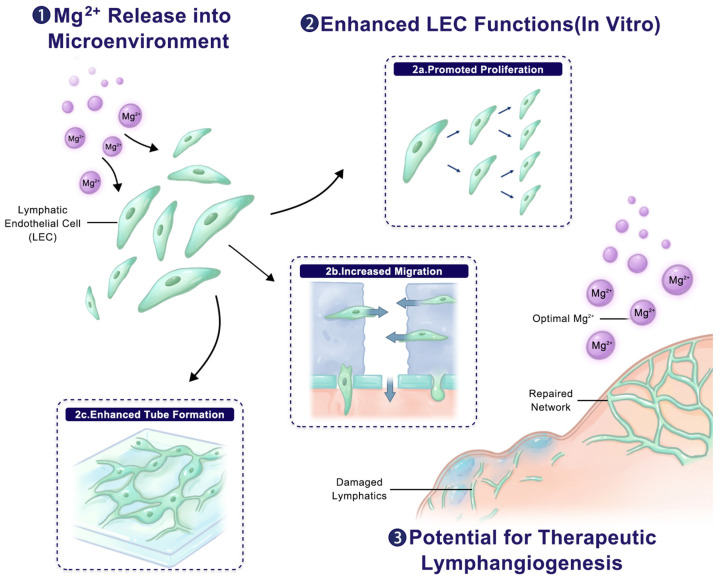
Schematic illustration of how an Mg^2+^-enriched extract microenvironment is associated with lymphatic endothelial cell (LEC) functional responses in vitro. (**1**) Mg^2+^ release: Mg^2+^ released from magnesium powders contributes to the ionic microenvironment in the culture system. (**2**) LEC functional responses in vitro: Appropriate extract conditions are associated with enhanced LEC proliferation (2a), migration (2b), and tube formation (2c). (**3**) Potential application relevance: These in vitro findings support the potential of Mg-based biomaterials to modulate lymphangiogenesis-related LEC behaviors and may inform future studies on lymphatic tissue repair.

The present study therefore systematically examines the impact of Mg^2+^ on key functional behaviors of lymphatic endothelial cells in vitro, utilizing extracts from biodegradable magnesium powder to simulate the degradation microenvironment. We assessed dose-dependent effects on LEC viability, proliferation, migration, and tube/network formation, while exploring potential underlying molecular mechanisms. To date, direct experimental evidence regarding the effects of Mg^2+^ on lymphatic endothelial cells remains limited. These findings are expected to provide foundational evidence for the lymphangiogenesis-related effects of Mg^2+^ on LEC and may inform the rational design of Mg-based biomaterials for lymphatic tissue repair. The central concept of this study, illustrating the association between an Mg^2+^-enriched extract microenvironment and enhanced LEC functional behaviors in vitro, is summarized in Scheme 1.

## 2. Materials and Methods

### 2.1. Morphological and Elemental Characterization of Magnesium Powder

The morphology and surface microstructure of the high-purity (99.9%) atomized spherical magnesium powder (Tangshan Weihao Magnesium Powder Co., Ltd., Tangshan, China) were characterized by scanning electron microscopy (SEM), and the elemental composition was further analyzed by energy-dispersive X-ray spectroscopy (EDS). Before characterization, magnesium powders were vacuum-dried, evenly dispersed on conductive carbon tape, and gently blown to remove loosely attached particles. SEM images were acquired using a scanning electron microscope (SEM3200, CIQTEK Co., Ltd., Hefei, China) at an accelerating voltage of 10 kV. For EDS analysis, elemental spectra and mapping were collected on representative powders using an EDS detector coupled to the SEM (Oxford Instruments, Abingdon, UK).

### 2.2. Preparation of Extracts

To simulate the degradation behavior of magnesium powder under in vitro culture conditions and obtain a magnesium ion-containing extract, the biodegradable magnesium material was added to basal Dulbecco’s Modified Eagle Medium (DMEM; Gibco, Grand Island, NY, USA) at 10 mg/mL and incubated at 37 °C with 5% CO_2_ on an orbital shaker with gentle agitation (50–80 rpm) to prevent particle sedimentation and agglomeration and to ensure sufficient contact between the material and the culture medium. The extraction was maintained for 24 h. After extraction, the supernatant was collected and filtered through a 0.22 μm sterile membrane filter to remove residual solid particles and to ensure sterility, yielding a clear stock extract.

### 2.3. Ion Release of Mg Material

The concentration of Mg^2+^ in the magnesium-based extracts was quantitatively determined using inductively coupled plasma mass spectrometry (ICP-MS, Agilent 7700, Agilent Technologies, Santa Clara, CA, USA). The stock extract and corresponding diluted samples were appropriately diluted according to instrument requirements prior to analysis. Based on the ICP-MS results, the undiluted stock extract (Mg) and a series of working extracts, including Mg (1:2), Mg (1:5), Mg (1:7.5), Mg (1:10), Mg (1:100), and Mg (1:1000), were freshly prepared using basal DMEM. Basal DMEM without extract served as the Control.

### 2.4. Cell Culture

Mouse lymphatic endothelial cells (LECs; SVEC4-10, Shanghai Baiyi Biotechnology Center, Shanghai, China) were cultured in DMEM (Gibco) supplemented with 10% fetal bovine serum (FBS; Gibco, Grand Island, NY, USA) and 1% penicillin/streptomycin (100 U/mL penicillin and 100 μg/mL streptomycin; Gibco, Grand Island, NY, USA) at 37 °C in a humidified incubator with 5% CO_2_. Cells were passaged routinely and used at consistent passage numbers across experiments.

### 2.5. Immunofluorescence Staining

Cells were fixed with 4% paraformaldehyde (Biosharp Life Sciences, Hefei, China), permeabilized with 0.1% Triton X-100 (Beyotime Biotechnology, Shanghai, China), and blocked with 5% BSA. The samples were incubated overnight at 4 °C with primary antibodies against LYVE1 (R&D Systems, Minneapolis, MN, USA) and PROX1 (Abcam, Cambridge, UK), followed by incubation with fluorophore-conjugated secondary antibodies for 1 h at room temperature in the dark. Nuclei were counterstained with DAPI (Beyotime Biotechnology, Shanghai, China). Images were captured using a fluorescence microscope (Leica Microsystems GmbH, Wetzlar, Germany), and cell identity was evaluated according to marker expression and cellular morphology.

### 2.6. Cell Counting Kit-8 (CCK-8) Assay

LECs were seeded in 96-well plates at an appropriate density and allowed to adhere overnight. Cells were then treated with Control, Mg, Mg (1:2), Mg (1:5), Mg (1:7.5), Mg (1:10), Mg (1:100), or Mg (1:1000) (prepared in basal DMEM as described above). At the indicated time points, 10 μL of CCK-8 reagent (Cell Counting Kit-8; Vazyme Biotech Co., Ltd., Nanjing, China) was added to each well containing 90 μL medium, followed by incubation at 37 °C for 2 h. Absorbance was measured at 450 nm using a microplate reader. Results were calculated based on optical density (OD) values and presented as relative proliferation.

### 2.7. Live/Dead Staining

To evaluate cell viability under different treatment conditions, a live/dead cell double staining assay was performed using Calcein-AM and propidium iodide (PI) (Calcein-AM/PI Double Staining Kit, Dojindo Laboratories, Kumamoto, Japan). LECs were treated with Control, Mg (1:10), Mg (1:100), or Mg (1:1000) for 24 h. The culture medium was then removed, and cells were washed with PBS to eliminate residual medium. Cells were incubated with Calcein-AM/PI working solution at 37 °C in the dark for 30 min. After incubation, cells were washed with PBS to remove excess dye and observed under an inverted fluorescence microscope. Live cells exhibited green fluorescence, whereas dead cells exhibited red fluorescence. Images were captured from randomly selected fields using an inverted fluorescence microscope for qualitative observation. Fluorescence images were further subjected to quantitative analysis of live/dead signals using ImageJ software (version 1.54, National Institutes of Health, Bethesda, MD, USA).

### 2.8. Scratch Assay

A scratch assay was performed to further evaluate cell migration. LECs were seeded into 24-well plates and cultured until reaching confluence. A sterile 200 μL pipette tip was used to create a linear scratch in the cell monolayer. The detached cells were removed by gentle washing, and the cells were then cultured with Control, Mg (1:10), Mg (1:100), or Mg (1:1000). Cell migration into the wound area was observed and imaged at 0, 6, 12, and 24 h using an inverted microscope. The wound closure area was quantified using ImageJ software.

### 2.9. Transwell Migration Assay

For the Transwell migration assay, 600 µL of DMEM containing the indicated extracts was added to the lower chamber of 24-well Transwell inserts (8 µm pore-size membranes; Corning Incorporated, Corning, NY, USA), and 100 µL of the cell suspension was seeded into the upper chamber. After incubation for 24 h at 37 °C with 5% CO_2_, cells on the inserts were fixed with 4% paraformaldehyde (PFA; Biosharp) for 30 min. Non-migrated cells on the upper surface were gently removed with cotton swabs, and migrated cells were stained with 0.5% crystal violet (Tianjin Zhiyuan Chemical Reagent Co., Ltd., Tianjin, China) for 30 min. Images were captured from five randomly selected fields per membrane using an inverted microscope (Leica Microsystems, Mannheim, Germany).

### 2.10. Tube Formation Assay

A tube formation assay was performed to assess the effects of magnesium-based extracts on the ability of LEC to form lymphatic-like networks. Matrigel (Corning Incorporated, Corning, NY, USA) was added to 24-well plates and allowed to polymerize at 37 °C. Subsequently, LECs were seeded onto the Matrigel-coated wells. Tube-like structures were observed under an inverted microscope at 2 h and 6 h after seeding. Quantitative analysis of tube formation, including total tube length and number of junctions, was performed using ImageJ software.

### 2.11. Intracellular ROS Assay

Intracellular reactive oxygen species (ROS) levels were measured using a DCFH-DA fluorescent probe (ROS Assay Kit, Dojindo Laboratories, Kumamoto, Japan). After treatment with Control, Mg (1:10), Mg (1:100), or Mg (1:1000) for 24 h, cells were washed twice with PBS. Cells were then incubated with DCFH-DA working solution at 37 °C in the dark for 30 min. Following incubation, cells were washed with PBS to remove excess probe. Images were captured from randomly selected fields using an inverted fluorescence microscope for qualitative observation and quantitative analysis.

### 2.12. RNA Extraction and qRT-PCR

Total RNA was extracted with Trizol reagent following the manufacturer’s protocol, then reverse-transcribed using the PrimeScript^®^ RT reagent kit with gDNA Eraser (TaKaRa Bio Inc., Kusatsu, Shiga, Japan), and analyzed by qRT-PCR using the SYBR Green PCR kit (TaKaRa Bio Inc., Kusatsu, Shiga, Japan) on a CFX96 system (Bio-Rad Laboratories, Hercules, CA, USA). GAPDH was used as the internal reference gene, and relative gene expression was calculated using the 2^−ΔΔCt^ method.

### 2.13. Western Blot Analysis

LECs were treated with Control, Mg (1:10), Mg (1:100), or Mg (1:1000) extracts for the indicated times. After treatment, cells were washed twice with ice-cold PBS and lysed in RIPA buffer supplemented with protease inhibitors on ice. The lysates were centrifuged at 12,000× *g* for 15 min at 4 °C, and the supernatants were collected for protein analysis. Equal amounts of protein were mixed with loading buffer, denatured at 95 °C for 5 min, separated by sodium dodecyl sulfate-polyacrylamide gel electrophoresis (SDS-PAGE), and transferred onto PVDF membranes (MilliporeSigma, Burlington, MA, USA). The membranes were blocked with 5% skim milk for 1 h at room temperature and incubated with primary antibodies at 4 °C overnight. After washing with TBST, the membranes were incubated with HRP-conjugated secondary antibodies for 1 h at room temperature. Protein bands were detected using an enhanced chemiluminescence (ECL) substrate (Tanon Science & Technology Co., Ltd., Shanghai, China) and visualized using a chemiluminescence image analysis system (Tanon). GAPDH and β-actin were used as internal loading controls for normalization. The primary antibodies used in this study included anti-VEGFA rabbit polyclonal antibody (ZEN-BIOSCIENCE Co., Ltd., Chengdu, China), anti-VEGFC rabbit polyclonal antibody (ZEN-BIOSCIENCE Co., Ltd., Chengdu, China), anti-VEGFR3 rabbit polyclonal antibody (Affinity Biosciences, Shanghai, China), anti-GAPDH rabbit polyclonal antibody (Affinity Biosciences, Shanghai, China), and anti-β-actin rabbit polyclonal antibody (Affinity Biosciences, Shanghai, China).

### 2.14. Statistical Analysis

All quantitative experiments were performed with at least three independent biological replicates. Data are presented as mean ± SD and analyzed using GraphPad Prism 10. Two-group comparisons were performed using an unpaired two-tailed Student’s *t*-test. Multiple-group comparisons were analyzed by one-way ANOVA with Tukey’s post hoc test, and time-course data were analyzed by two-way ANOVA. Statistical significance was defined as *p* < 0.05 (*), *p* < 0.01 (**), *p* < 0.001 (***), *p* < 0.0001 (****) and *p* > 0.05 (ns).

## 3. Results and Discussion

### 3.1. Morphological and Elemental Characterization of Magnesium Powder

**Figure 1:**
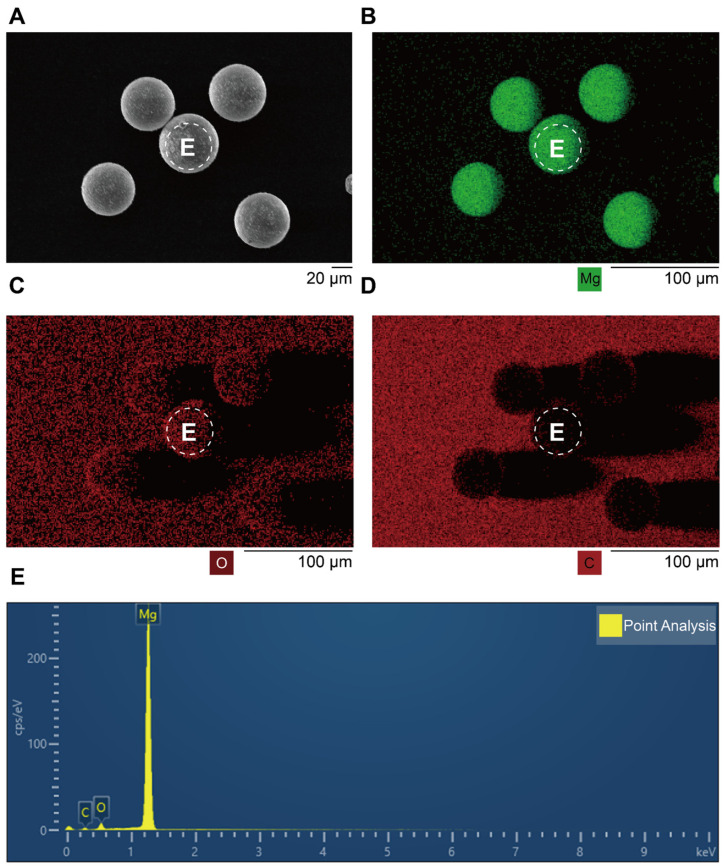
Morphological and elemental characterization of magnesium powder particles. (**A**) Representative scanning electron microscopy (SEM) image showing the spherical morphology and surface microstructure of the magnesium powder particles. Scale bars = 20 μm. (**B**–**D**) Energy-dispersive X-ray spectroscopy (EDS) elemental mapping of representative particles, showing the spatial distribution of Mg (**B**), O (**C**), and C (**D**) signals. Scale bars = 100 μm. (**E**) Representative EDS point-analysis spectrum of a selected particle region, showing characteristic Mg peaks together with O (and minor C) signals.

As shown in Figure 1A, SEM images demonstrated that the commercial magnesium powder particles exhibited a relatively regular spherical morphology with overall uniform features. Most particles appeared intact, with no obvious collapse or severe fracture observed in the imaged fields. Visual inspection of the SEM images (scale bar: 100 μm) indicated that the particle size ranged primarily from approximately 30 to 100 μm, although some interparticle variation was present. These morphological features support the suitability of the powder as a source material for subsequent extraction-based experiments [30,31].

Figure 1B–D present the EDS elemental mapping results, showing the spatial distribution of Mg, O, and C signals in representative particles. The Mg signal was predominantly localized to the particles, confirming that the analyzed regions were magnesium-rich. In addition, O signals were observed across the particle surfaces, which is consistent with the presence of surface oxidation and suggests the formation of a surface oxide/passivation layer. By contrast, the C signal was comparatively weak and was mainly attributed to background and/or sample-mounting-related contributions during EDS acquisition. Furthermore, the EDS point spectrum of the selected region (Figure 1E) displayed characteristic Mg peaks together with O and minor C signals, which was in agreement with the mapping results. Collectively, these data confirm the expected morphology and elemental features of the magnesium powder and provide a characterization basis for the subsequent ion-release quantification described in Section 3.2.

### 3.2. Magnesium Ion Release from Magnesium Powder and Dilution Design for In Vitro Evaluation

ICP-MS analysis was performed to quantify Mg^2+^ release from the magnesium powder extract after 24 h of extraction. The undiluted stock extract contained 284.54 mg/L Mg^2+^ (≈11.71 mM), whereas fresh DMEM showed a basal Mg^2+^ concentration of 15.02 mg/L (≈0.62 mM). Based on the ICP-MS results, the stock extract was diluted with fresh DMEM to prepare a series of working extracts, including Mg, Mg (1:2), Mg (1:5), Mg (1:7.5), Mg (1:10), Mg (1:100), and Mg (1:1000), for cell viability screening. The final Mg^2+^ concentrations in these conditions were calculated to be 284.54 mg/L (≈11.71 mM), 149.78 mg/L (≈6.16 mM), 68.92 mg/L (≈2.84 mM), 50.96 mg/L (≈2.10 mM), 41.97 mg/L (≈1.73 mM), 17.72 mg/L (≈0.73 mM), and 15.29 mg/L (≈0.63 mM), respectively. Basal DMEM without extract served as the Control.

In addition, the pH values of the extract-containing media were measured. The undiluted Mg extract showed a relatively alkaline pH of approximately 9.1, whereas the pH values of Mg (1:10), Mg (1:100), and Mg (1:1000) were approximately 7.8, 7.7, and 7.6, respectively, compared with 7.6 in the Control medium. These results indicate that dilution substantially reduced the alkalinity of the extract and brought the selected working concentrations close to the basal medium condition.

This dilution strategy generated a concentration gradient ranging from near-basal to moderately elevated and high Mg^2+^ levels in DMEM, enabling the evaluation of concentration-dependent LEC responses under an extract-derived ionic microenvironment [26,32,33]. The 1:1000 dilution approximated the basal Mg^2+^ level of fresh DMEM and served as a near-baseline reference condition, whereas the 1:10 and 1:100 dilutions provided moderately elevated Mg^2+^ exposure conditions for subsequent functional assays. Higher-concentration groups were included in the viability assay to assess cellular tolerance under relatively high Mg^2+^ exposure. Together, these conditions enabled the assessment of dose-dependent changes in LEC responses to the magnesium-enriched extract.

### 3.3. Cytocompatibility and Proliferative Effects of Magnesium Extracts on LECs

**Figure 2:**
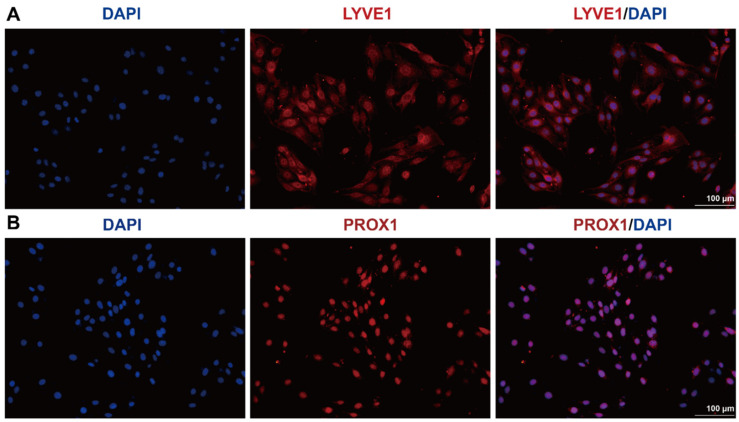
Immunofluorescence identification of cultured lymphatic endothelial cells (LECs). (**A**) Representative immunofluorescence images showing LYVE1 expression in cultured LECs. Nuclei were counterstained with DAPI. (**B**) Representative immunofluorescence images showing PROX1 expression in cultured LECs. Nuclei were counterstained with DAPI. Merged images demonstrate positive expression of LYVE1 and PROX1 in the cultured cells, supporting their lymphatic endothelial phenotype. Scale bars = 100 μm.

As shown in Figure 2, the cultured cells exhibited positive immunofluorescence staining for LYVE1 and PROX1, two representative markers of lymphatic endothelial cells. PROX1 was mainly localized in the nucleus, while LYVE1 staining was clearly observed in the cultured cells. These findings support the lymphatic endothelial identity of the cultured cells used in the present study.

**Figure 3:**
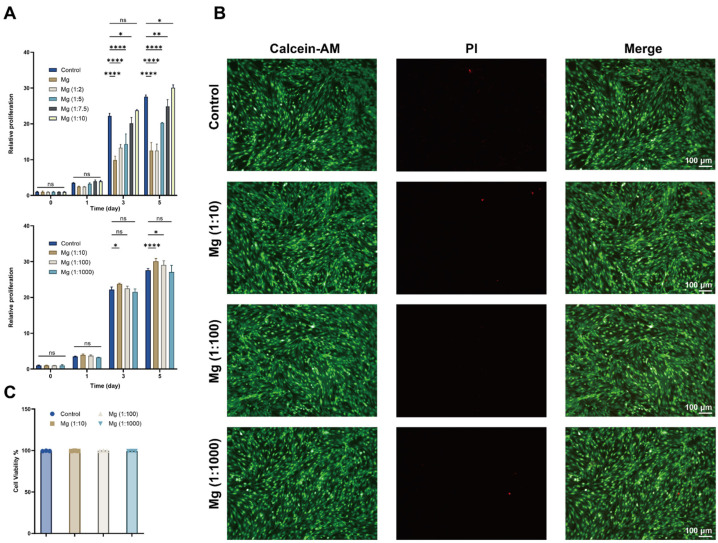
Effects of magnesium extracts on LEC viability and proliferation. (**A**) CCK-8 assay showing the effects of different magnesium extract concentrations on LEC proliferation at the indicated time points. Upper panel: screening of Control, Mg, Mg (1:2), Mg (1:5), Mg (1:7.5), and Mg (1:10) groups. Lower panel: comparison among the selected working concentrations Control, Mg (1:10), Mg (1:100), and Mg (1:1000). (**B**) Representative Live/Dead staining images of LECs treated with Control, Mg (1:10), Mg (1:100), and Mg (1:1000) extracts. Live cells are stained green with Calcein-AM, and dead cells are stained red with PI. (**C**) Quantitative analysis of cell viability based on Live/Dead fluorescence staining. Data are presented as the mean ± SEM. * *p* < 0.05, ** *p* < 0.01, **** *p* < 0.0001; ns, not significant. Scale bars = 100 μm.

The cytocompatibility and proliferative effects of the magnesium extracts on LECs were evaluated using CCK-8 assays and Live/Dead staining. As shown in Figure 3A, the initial CCK-8 screening revealed a concentration-dependent effect of the magnesium extract on LEC proliferation. Compared with the Control group, the higher-concentration groups, including Mg, Mg (1:2), Mg (1:5), and Mg (1:7.5), generally showed lower proliferation levels, whereas Mg (1:10) exhibited a more favorable effect and exceeded the control at later time points. Consistent with the above physicochemical characterization, the reduced proliferative support observed in the undiluted or less diluted extract groups may be associated, at least in part, with the relatively more alkaline microenvironment. Based on this preliminary screening, Mg (1:10), Mg (1:100), and Mg (1:1000) were selected for subsequent experiments.

Further comparison among these selected concentrations showed that Mg (1:10) and Mg (1:100) promoted LEC proliferation more effectively than the near-basal condition Mg (1:1000), while Mg (1:1000) remained close to the control level (Figure 3A). The proliferative effects of Mg (1:10) and Mg (1:100) were generally comparable at several time points, indicating that moderately elevated Mg^2+^ exposure was more favorable for LEC growth than either near-basal or excessively high exposure conditions.

These quantitative results were further supported by the Live/Dead staining results. As shown in Figure 3B, all tested groups exhibited predominantly Calcein-AM-positive green fluorescence with only sparse PI-positive red signals, indicating good cytocompatibility under the selected diluted extract conditions. Consistently, quantitative analysis of fluorescence staining (Figure 3C) showed no obvious reduction in cell viability among the Control, Mg (1:10), Mg (1:100), and Mg (1:1000) groups.

Taken together, these results indicate that the magnesium extracts showed good cytocompatibility toward LECs within the tested dilution range. Among the tested conditions, Mg (1:10) showed the most favorable proliferative effect in the initial screening, while Mg (1:10) and Mg (1:100) were more beneficial for LEC growth than Mg (1:1000) in the subsequent comparison. These findings provided the basis for selecting the working concentrations for the following functional assays.

### 3.4. Magnesium Extracts Accelerate LEC Migration In Vitro

Endothelial cell migration is an important process in vascular remodeling and lymphatic regeneration. To evaluate the pro-migratory effects of magnesium extracts on LEC, we conducted both scratch wound healing and Transwell migration assays [34,35,36].

**Figure 4:**
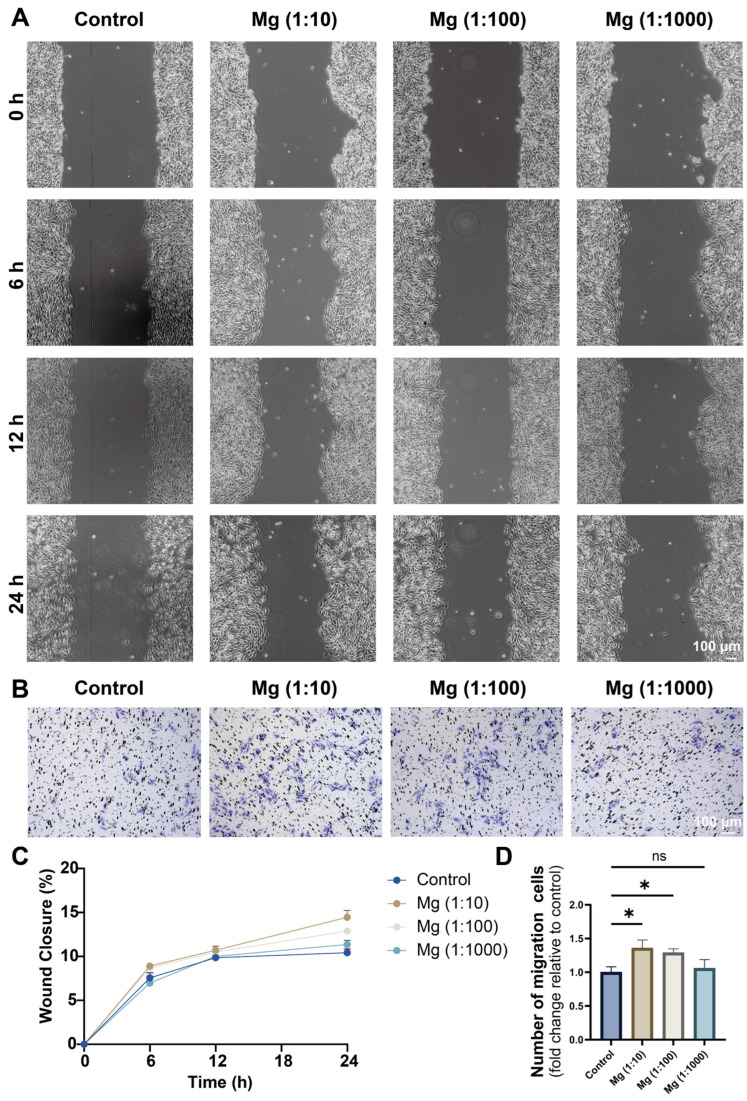
Magnesium extract promotes LEC migration in vitro. (**A**) Representative images of the scratch wound healing assay at 0, 6, 12, and 24 h in lymphatic endothelial cells (LECs) treated with fresh DMEM (Control) or magnesium extract diluted with DMEM at the indicated ratios [Mg (1:10), Mg (1:100), and Mg (1:1000)]. Scale bar: 100 μm. (**B**) Representative images of the Transwell migration assay (crystal violet staining) after 24 h under the same treatment conditions. Scale bar: 100 μm. (**C**) Quantification of wound closure over time. (**D**) Quantification of migrated cells, expressed as fold change relative to the control group. Data are presented as mean ± SD (*n* = 3 independent biological replicates). Statistical analysis for (**C**) was performed using two-way ANOVA (group × time), followed by Tukey’s multiple-comparisons test. Statistical analysis for (**D**) was performed using one-way ANOVA followed by Tukey’s multiple-comparisons test. Statistical significance: ns, not significant; * *p* < 0.05 (as indicated).

As shown in Figure 4A, the scratch assay revealed a time-dependent acceleration of wound closure in the Mg (1:10) and Mg (1:100) groups compared with the control. Quantitative analysis confirmed that both dilutions significantly enhanced LEC motility over 6–24 h (Figure 4C). Notably, the Mg (1:10) group exhibited a stronger pro-migratory effect than the Mg (1:100) group at the indicated time points. In contrast, the Mg (1:1000) group showed limited changes, with migration rates remaining comparable to basal levels (Figure 4A,C).

These observations were further supported by Transwell assays, which assess directional cell migration. Representative images showed an increased number of migrated LEC in the Mg (1:10) and Mg (1:100) groups relative to the control (Figure 4B). Consistent with the scratch assay, quantitative analysis demonstrated significantly elevated numbers of migrated cells in both extract groups, with the Mg (1:10) group again showing the strongest response (Figure 4D). Conversely, the Mg (1:1000) group exhibited limited enhancement and remained close to control values.

These results show that magnesium extracts promote LEC migration within the tested dilution range. Both the scratch assay (lateral migration) and the Transwell assay (directional migration) showed the same trend, which strengthens the evidence for a pro-migratory effect and suggests that the result is not dependent on a single assay format. When considered together with the proliferation data in Section 3.3, the findings further indicate that appropriately diluted magnesium extracts, especially the 1:10 dilution, provide a favorable microenvironment for multiple LEC behaviors associated with lymphangiogenesis-related responses in vitro. This may reflect that moderately elevated Mg^2+^ conditions provide a more favorable microenvironment for LEC motility than either near-basal or higher-concentration conditions.

### 3.5. Magnesium Extracts Enhance Tube Formation and Network Reorganization in LECs In Vitro

**Figure 5:**
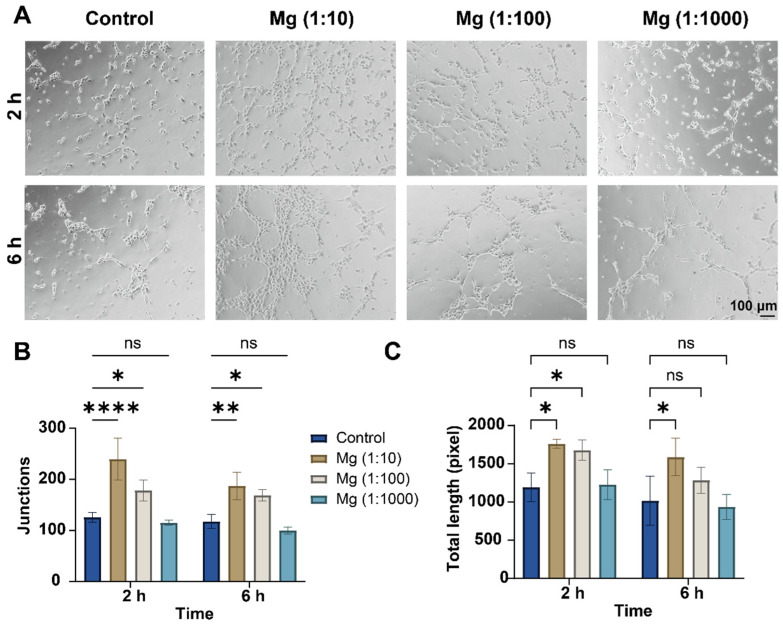
Magnesium extract enhances in vitro tube formation of lymphatic endothelial cells. (**A**) Representative bright-field images of LEC tube formation on Matrigel at 2 h and 6 h after treatment with fresh DMEM (Control) or magnesium extract diluted with DMEM at the indicated ratios [Mg (1:10), Mg (1:100), and Mg (1:1000)]. (**B**,**C**) Quantification of network formation, including the number of junctions (**B**) and total tube length (**C**). Data are presented as mean ± SD (*n* = 3 independent biological replicates). For each time point (2 h or 6 h), statistical analysis in (**B**,**C**) was performed using one-way ANOVA followed by Tukey’s multiple-comparisons test. Statistical significance is indicated as ns, not significant; * *p* < 0.05, ** *p* < 0.01, **** *p* < 0.0001 (as shown). Scale bar, as indicated.

To further evaluate lymphangiogenesis-related endothelial behavior, we performed an in vitro tube formation assay, which mimics the reorganization of cells into capillary-like networks [35,36,37]. Control LEC formed relatively sparse and partially disconnected structures at both 2 h and 6 h (Figure 5A). In contrast, Mg (1:10) and Mg (1:100) promoted more robust and interconnected networks, characterized by improved continuity and more closed-loop structures (Figure 5A). Quantitative analysis confirmed that both total tube length and junction number were significantly increased in Mg (1:10) and Mg (1:100) compared with the control, whereas Mg (1:1000) generally produced network patterns comparable to the control (Figure 5A–C).

These results indicate that appropriately diluted magnesium extracts enhance the structural reorganization capacity of LEC during network formation. The improved continuity and increased junction formation in the Mg (1:10) and Mg (1:100) groups are consistent with the enhanced migratory activity observed in Section 3.4, since tube assembly requires coordinated cell migration and cell–cell interaction. The similar trend observed in migration and tube formation suggests that the beneficial effects of magnesium extract are not restricted to a single functional readout. Together, these findings support a positive effect of a magnesium ion-based microenvironment on lymphangiogenesis-related endothelial behavior in vitro.

### 3.6. Magnesium Extracts Are Associated with Reduced Intracellular ROS Levels in LEC

**Figure 6:**
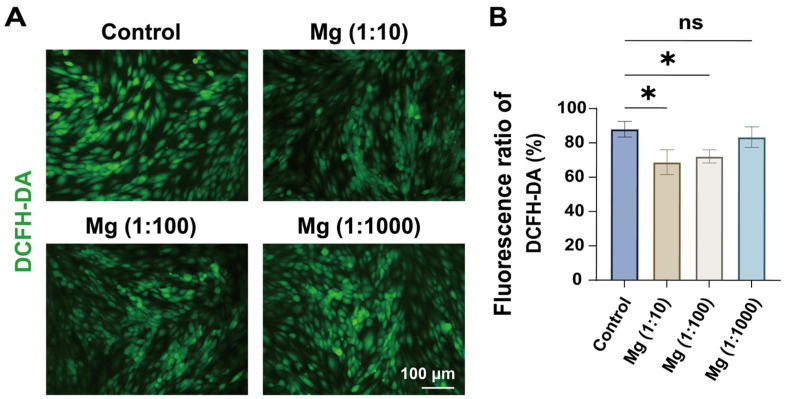
Intracellular ROS levels in LEC after treatment with magnesium extract dilutions. (**A**) Representative fluorescence images of DCFH-DA staining in lymphatic endothelial cells (LECs) cultured in fresh DMEM (Control) or magnesium extract diluted with DMEM at 1:10, 1:100, or 1:1000 [Mg (1:10), Mg (1:100), Mg (1:1000)]. Scale bar: 100 μm. (**B**) Quantification of DCFH-DA fluorescence intensity (normalized to the control group, as indicated). Data are presented as mean ± SD (*n* = 3 independent biological replicates). Statistical analysis for (**B**) was performed using one-way ANOVA followed by Tukey’s multiple-comparisons test. Statistical significance: ns, not significant; * *p* < 0.05.

Having established the superior cellular behaviors, we next investigated potential microenvironment-related changes, focusing on oxidative stress regulation. Oxidative stress is a major barrier hindering regeneration and can impair endothelial survival and function [38,39]. We detected intracellular ROS levels using DCFH-DA staining. As shown in Figure 6, representative fluorescence images showed reduced DCF signal intensity in Mg (1:10) and Mg (1:100) compared with the control (Figure 6A). Quantification confirmed that intracellular ROS levels were significantly lower in Mg (1:10) and Mg (1:100) versus the control, whereas Mg (1:1000) showed limited change and remained close to the control (Figure 6A,B).

These results indicate that appropriately diluted magnesium extracts are associated with reduced intracellular ROS accumulation in LEC under the present culture conditions. The stronger ROS-lowering effects observed in the Mg (1:10) and Mg (1:100) groups are consistent with their superior performance in proliferation/viability, migration, and tube formation (Section 3.3, Section 3.4 and Section 3.5), suggesting that improved redox homeostasis may contribute to the pro-lymphangiogenic/pro-regenerative LEC phenotype. This association provides a plausible microenvironment-related explanation for the superior cellular responses observed in the Mg (1:10) and Mg (1:100) groups. Although the current data do not establish a direct ROS-scavenging mechanism, they support the view that magnesium-derived ionic cues help create a more favorable, low-stress microenvironment for LEC functional responses [38,39].

### 3.7. Magnesium Extracts Are Associated with Activation of VEGF-Related Molecular Responses

**Figure 7:**
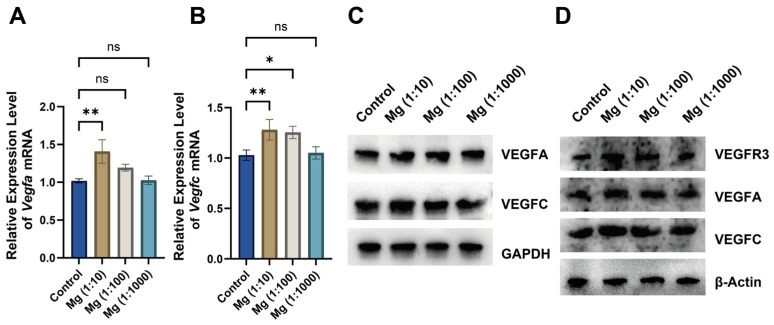
Effects of magnesium extracts on lymphangiogenesis-related gene and protein expression in LECs. (**A**) Relative Vegfa mRNA expression levels in LECs treated with Control, Mg (1:10), Mg (1:100), and Mg (1:1000), as determined by qRT-PCR. (**B**) Relative Vegfc mRNA expression levels in LECs under the same treatment conditions. (**C**) Representative Western blot images showing VEGFA and VEGFC protein expression in LECs after treatment with different concentrations of magnesium extracts, with GAPDH used as the internal loading control. (**D**) Additional Western blot analysis showing VEGFR3 protein expression under the same treatment conditions, with β-actin used as the internal loading control. Data in (**A**,**B**) are presented as the mean ± SD (*n* = 3). Statistical analysis for (**A**,**B**) was performed using one-way ANOVA followed by Tukey’s multiple-comparisons test. Statistical significance is indicated as * *p* < 0.05, ** *p* < 0.01; ns, not significant.

Finally, to further explore molecular changes associated with the enhanced migration and tube formation, we analyzed the expression of lymphangiogenesis-related factors by qRT-PCR (Table 1) and Western blot. Vascular endothelial growth factor (VEGF) signaling is a pivotal regulatory axis in vascular biology, and VEGF-C-associated signaling is widely recognized as a core driver of lymphangiogenesis [40,41,42]. Compared with the control, Mg (1:10) significantly increased VEGFA and VEGFC expression (Figure 7A,B). Mg (1:100) also significantly upregulated VEGFC transcripts relative to the control and showed an elevated trend in VEGFA, whereas Mg (1:1000) remained close to the control level (Figure 7A,B). These transcriptional changes are consistent with the enhanced migration and tube formation phenotypes observed under Mg (1:10) and Mg (1:100) conditions (Figure 4 and Figure 5).

At the protein level, Western blot analysis showed that VEGFA and VEGFC expression was increased in the Mg (1:10) and Mg (1:100) groups compared with the Control and Mg (1:1000) groups (Figure 7C,D). In addition, the expression of VEGFR3, a key receptor involved in lymphangiogenic signaling, was also elevated in the Mg (1:10) and Mg (1:100) groups, particularly in the Mg (1:10) group, whereas Mg (1:1000) showed no obvious increase relative to the control (Figure 7D). These molecular changes were in line with the pro-lymphangiogenic phenotypes induced by magnesium extracts in vitro.

Taken together, these findings suggest that magnesium extracts, especially under the Mg (1:10) and Mg (1:100) conditions, promote lymphangiogenesis-related behaviors in LECs, accompanied by upregulation of VEGFA, VEGFC, and VEGFR3. These results support the involvement of VEGF-related signaling in the biological response of LECs to magnesium extract treatment. However, changes in mRNA and protein expression alone are insufficient to establish pathway dependency or causal activation. In line with this, Mg^2+^ has been implicated in signaling pathways related to endothelial survival and migration, including PI3K/AKT and MAPK signaling [24,27,28,29], which may serve as plausible mechanistic entry points for future studies. Overall, the present findings support magnesium-derived ionic cues as a bioactive stimulus that enhances lymphangiogenesis-related behaviors of LECs in vitro and provide a basis for subsequent in vivo and mechanistic validation [43].

### 3.8. Limitations and Future Perspectives

Although the present study provides evidence that magnesium-derived extracts can enhance lymphatic endothelial cell functions associated with lymphangiogenesis, several aspects should be considered when interpreting these findings. The experimental system is limited to in vitro conditions, which do not fully recapitulate the complexity of the in vivo lymphatic microenvironment, where multicellular interactions and systemic regulatory factors may influence lymphangiogenic processes. In addition, the current results primarily demonstrate associations between Mg^2+^ treatment, reduced intracellular oxidative stress, and increased VEGF expression, without establishing direct causal relationships or identifying the upstream signaling mechanisms mediating these effects. While candidate signaling pathways, including PI3K/Akt and MAPK/ERK, may potentially be involved in mediating the phenotypic and functional responses of LECs to Mg^2+^, this remains speculative and requires explicit verification in future studies. Furthermore, the use of magnesium powder-derived extracts represents a degradation-associated ionic microenvironment, in which the observed biological responses may reflect the combined effects of Mg^2+^ and other physicochemical factors, rather than Mg^2+^ alone [44,45]. In addition, the present study did not specifically characterize potential interactions between released Mg^2+^ and DMEM components, such as complex formation, precipitation, or aggregation, which may also influence the physicochemical properties of the extract-containing medium and thereby affect cellular responses.

From a translational perspective, while the promotion of lymphangiogenic activity may be beneficial for tissue repair and regeneration, its potential context-dependent effects should also be taken into account. In particular, although enhanced lymphangiogenesis may be beneficial in regenerative settings, it could also be undesirable in certain pathological contexts, such as cancer, where lymphatic vessel growth may potentially facilitate tumor dissemination or metastasis. Therefore, further studies incorporating pathway-specific analyses, well-controlled ionic systems, and in vivo models are required to better define the mechanisms, specificity, and safety profile of magnesium-based strategies. Collectively, the present findings provide an initial in vitro basis for understanding the role of magnesium in lymphatic biology and support further investigation into its potential applications in regenerative medicine.

## 4. Conclusions

This study provides evidence that Mg^2+^ ions released from biodegradable magnesium-based materials can enhance key lymphangiogenic behaviors of LECs in vitro. Within the tested dilution range, the magnesium extracts exhibited favorable cytocompatibility and promoted LEC proliferation, migration, and tube formation. The Mg (1:10) and Mg (1:100) groups showed more pronounced effects compared with the Mg (1:1000) group, suggesting the presence of an optimal concentration range for biological activity. These functional changes were accompanied by reduced intracellular ROS levels and increased VEGFA/VEGFC expression, indicating that magnesium-derived ionic cues may be associated with a more favorable microenvironment for LEC functional responses. However, these observations reflect associative relationships rather than direct mechanistic evidence. Overall, these findings support the potential of biodegradable Mg-based materials as a promising, intrinsically bioactive platform for lymphatic tissue regeneration, with potential applications in treating post-surgical lymphedema, chronic wounds, and engineered vascularized tissues. Nevertheless, given that the present study is limited to in vitro conditions, further investigations are required to elucidate the underlying mechanisms and to validate the translational potential of these findings in vivo.

## Figures and Tables

**Table 1:** Primer sequences used for qRT-PCR (Mouse).

Gene	Forward Primer Sequence (5′-3′)	Reverse Primer Sequence (5′-3′)
*GAPDH*	ACTCCACTCACGGCAAATTC	TCTCCATGGTGGTGAAGACA
*VEGFA*	CTGCTGTAACGATGAAGCCCTG	GCTGTAGGAAGCTCATCTCTCC
*VEGFC*	GTGAGGTGTGTATAGATGTGGGG	ACGTCTTGCTGAGGTAACCTG

## Data Availability

The data presented in this study are included in the article. Further inquiries can be directed to the corresponding author.
